# Comparing Two Improved Techniques With the Traditional Surgical Techniques for Intra and Extramedullary Spinal Tumor Resection: A Report of 280 Cases

**DOI:** 10.3389/fsurg.2022.892470

**Published:** 2022-04-25

**Authors:** Kamaliddin Djumanov, Gayrat Kariev, Gennady Chmutin, Gennady Antonov, Egor Chmutin, Gerald Musa, Adam Maier, Alina Shumadalova

**Affiliations:** ^1^Republican Specialized Scientific and Practical Medical Center of Neurosurgery, Tashkent, Uzbekistan; ^2^Department of Neurosurgery, Tashkent Pediatric Medical Institute, Tashkent, Uzbekistan; ^3^Department of Nervous Diseases and Neurosurgery, Peoples' Friendship University of Russia (RUDN University), Moscow, Russia; ^4^Federal State Budgetary Institution of Medical Department of Moscow “Morozov Children's City Clinical Hospital of Medical Department of Moscow”, Moscow, Russia; ^5^3rd Central Military Clinical Hospital Named After A.A. Vishnevsky Under the Ministry of Defense of the Russian Federation, Krasnogorsk, Russia; ^6^Department of General Chemistry, Bashkir State Medical University, Ufa, Russia

**Keywords:** spinal cord tumors, syringohydromyelia, differentiated surgical tactics, intramedullary tumors, extramedullary tumors, Nurick score

## Abstract

**Objectives:**

Spinal tumors remain a challenging problem in modern neurosurgery. The high rate of postoperative morbidity associated with intramedullary tumors makes the need for safer surgical techniques invaluable. This study analyses our experience with the treatment of spinal cord tumors and compares traditional management and a new different surgical approach to intramedullary tumors with an associated hydrosyringomyelia.

**Materials and Methods:**

This retrospective study compared standard surgical techniques and 2 newer modified techniques for intra and extramedullary spinal tumors at the Neurosurgery center for spinal cord tumors of the Republic of Uzbekistan. Preoperative neurological status was recorded with the ASIA/ISNCSCI scale. Postoperative outcome was graded using the Nurrick score.

**Results:**

Of the 280 cases, there were 220 (78.5%) extramedullary and 60 (21.5%) with intramedullary spinal tumors. The control and main group had 159 (56.8%) and 121 (43.2%) patients, respectively. Severe compression myelopathy (ASIA- A, B, C) was 217 (77.5%) patients i.e., ASIA A-39 (13.9%); B-74 (26.4%), and C-104 (37.1%). In 74 extramedullary tumors (33.6%) treated with the new method, good postoperative outcomes in 44 cases (59.5%) with OR = 1.9; 95% CI 1.1–3.3 (*p* < 0.05). Thirty-seven (61.7%) intramedullary tumors were treated with the newer modified technique. There was no difference with the standard method (*p* = 0.15). However, when comparing postoperative Nurick grade 1–2 with grade 3–4, the newer strategy was superior with improvement in 24 (65%) patients, OR = 3.46; 95% CI 1.2–10.3 (*p* < 0.05).

**Conclusion:**

When compared with standard methods, the proposed newer modified strategy of surgical treatment of spinal cord tumors with the insertion of a syringosubarachnoid shunt in the presence of an associated hydrosyringomyelia is associated with better postoperative outcome (Nurick 1 and 2) in 64.8%.

## Introduction

The term “spinal cord tumors” includes all oncological processes in the region of the spinal column. They are generally classified as: intramedullary, extramedullary (emanating from the inner layer of the dura, dental ligament, pial membrane, intradural part of the spinal root), extradural tumors that are also further divided into primary tumors originating from the vertebra, periosteum, ligaments, cartilage, the outer layer of the dura mater and secondary (metastatic) tumors (1, 2). Spinal cord tumors account for 2% of all neoplasms, 3% of nervous system tumors, and 20% of tumors of the central nervous system in adults. The ratio of spinal to brain tumors is around 1: 9. Most often, spinal cord tumors are observed in the socially active group of people aged 30–50 years, which makes this problem one of great relevance in medicine today. However, surgery of spinal tumors is one of the most difficult problems of neurosurgery. Among the tumors of the spinal cord, according to modern authors, extramedullary tumors predominate and constitute up to 70% of all spinal tumors. The results of surgical treatment of spinal cord tumors depend on many factors: the duration of the disease, the extent of neurological deficit, the radicality of tumor removal, and the extent of intraoperative trauma to the spinal cord (3, 4). All these factors should be considered in combination (3, 5).

To date, the main method of treating spinal cord tumors is surgical excision. However, indications for specific surgical approaches to these tumors are still insufficiently developed and depend on the anatomical location of the tumor, histological diagnosis, and the aggressiveness of the neoplasm (3, 6, 7). Currently, the results of spinal cord tumor surgery are still unsatisfactory, and far from perfect (8). The high rate of postoperative morbidity calls for the need for further research aimed at improving the results of spinal tumors treatment. This study analyses our experience with the treatment of spinal cord tumors and compares traditional management and a new different surgical approach to intramedullary tumors with an associated hydrosyringomyelia.

## Materials and Methods

### Study Design, Setting, and Participants

This was a retrospective study. Case files of all patients with surgically managed spinal tumors between 2014 and 2021 were extensively analyzed. All patients were managed at the Republican Specialized Scientific and Practical Medical Center of Neurosurgery in Tashkent, Uzbekistan during this period. The patients were divided into two groups i.e., the control and the main group depending on the when the patients were managed and the procedure used. The control group included patients treated according to standard traditional tumor resection strategies between 2014 and 2017. The main study group included patients managed using a newer modified strategy between 2018 and 2021 at the Neurosurgery center for spinal cord tumors of the Republic of Uzbekistan.

The newer modified surgical strategy were basically improvements on traditional methods of removing tumors of extra- and intramedullary localization. For extramedullary tumors, the essence of the method is to conduct internal decompression of the tumor, to minimize traumatization of the spinal cord and main vessels. In this method, the intracapsular part is removed in small fractions. The tumor debulking begins with the areas farthest from the great vessels, the roots of the spinal cord, and the spinal cord itself.

In the surgical treatment of intramedullary tumors associated with an intramedullary cyst, the newer modified strategy involved inserting a syringosubarachnoid bypass shunt after excision of intramedullary tumors. This is done to prevent the re-accumulation of cerebrospinal fluid and the expansion of the intramedullary dead space remaining after tumor excision.

**Figure 1:**
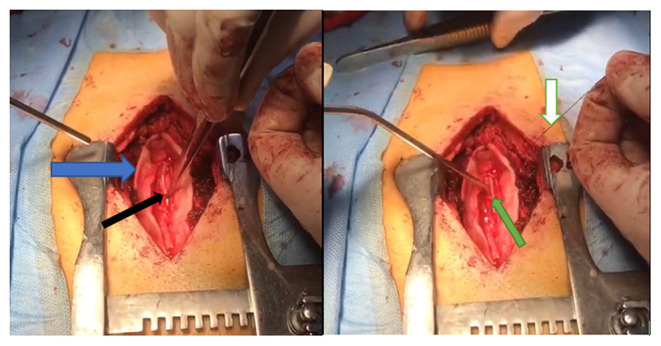
Intraoperative images showing the insertion of the syringosubarachnoid shunt. The dura is opened (blue arrow), the tube is inserted into the resection dead space and into the syrinx (black arrow). The shunt is seen laying in the resection dead space is anchored to the overlying dura using a 6-0 silk suture (white arrow).

A 6–8 cm piece is fashioned from the standard 3mm silicone tubing used for ventriculoperitoneal shunts. Following the standard microsurgical approach and microsurgical tumor resection, the proximal end of the already prepared tube is inserted into the resection cavity and advanced either cranially or caudally depending on the location, into the syrinx cavity. Patency was confirmed by CSF egress through the tube. The distal end of the tube was placed in the subdural space and anchored to the overlying dura using a 6-0 silk suture to avoid shunt migration (Figure 1). The dura was closed in a water tight fashion using 6-0 polypropylene monofilament. The remainder of the closure was performed in a standard multilayer fashion for a spinal procedure.

**Figure 2:**
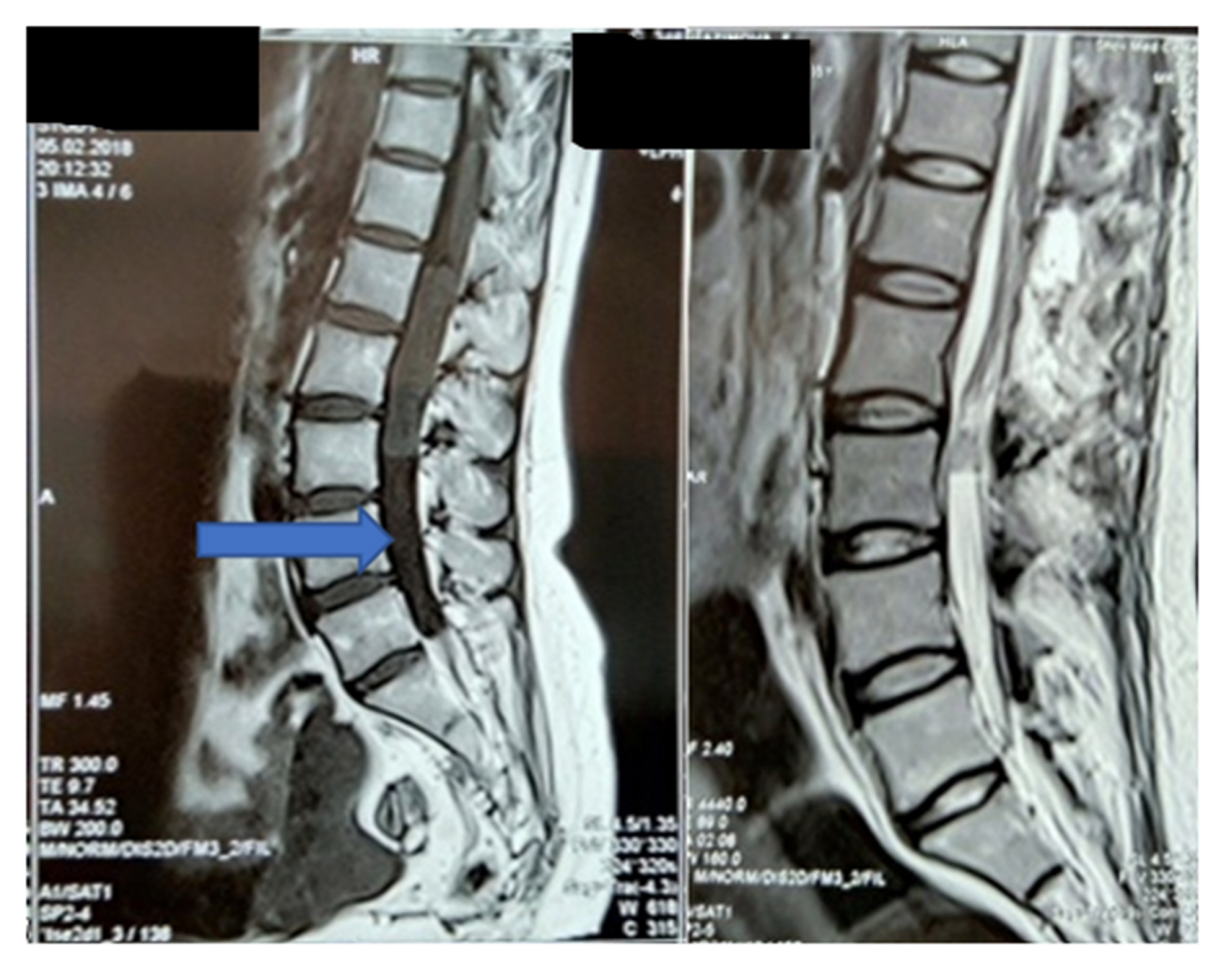
MRI of the cervical spine, preoperative and 2 days post-surgery. A 36-year-old with a Neuroma on the ventral surface of the spinal cord, at the level of C1-C2 (blue arrow). Control MRI shows total tumor removal using an improved method (orange arrow).

**Figure 3:**
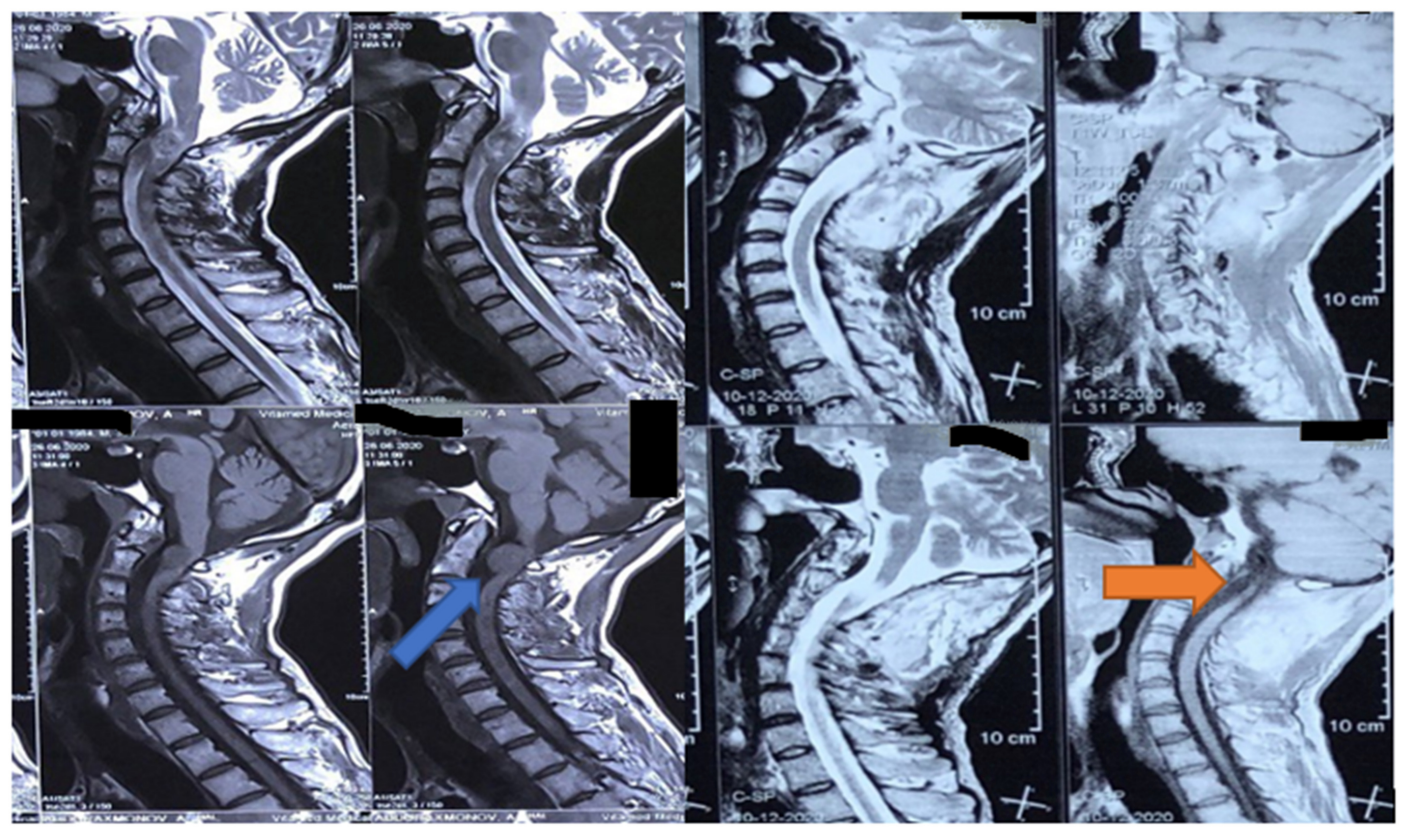
A pre-and post-surgery MRI of 32 years old with ependymoma of the roots of the cauda equina and syringomyelia between L3-L5 (blue arrow).

The indications for surgical intervention in the reviewed cases included; the mere presence of a tumor, compression of the spinal cord or its roots, and neurological deficit of varying severity. Figures 2, 3 illustrate examples of two extramedullary tumors managed in this study, a C1-C2 anterolateral neuroma and caudal equina ependymoma, respectively.

Preoperative severity of neurological deficit was graded according ASIA/ISNCSCI scale (2015). For a comparative assessment of the postoperative neurological status, the Nurick scale (NS) was used i.e., Grade 1- complete regression of neurological symptoms, grade 2- improvement, grade 3- No change, and grade 4- deterioration of neurological status.

### Statistical Analysis

The statistical analysis was carried out using the IBM SPSS 23 program. The Chi-squared criterion (χ^2^) was used to determine the presence of association, the strength of the association was determined using the odds ratio (OR) with a 95% confidence interval (95% CI). The significance level was set at *p* < 0.05, for all analyses.

## Results

The reviewed cases included 280 patients, 220 (78.5%) patients had extramedullary tumors and 60 patients (21.5%) with intramedullary spinal tumors. These different histological diagnoses were analyzed as shown in Table 1. In both groups, meningiomas and neuromas were the most common extramedullary tumors while ependymoma was the most frequent intramedullary tumor. The control group managed with standard techniques included 159 (56.8%) managed between 2014 and 2017 while the main study group included 121 (43.2%) managed with the modified methods between 2017 and 2021.

**Table 1:** Distribution of the spinal tumors by histological diagnosis.

**Histology**	**Control group**	**Main group**	**Total**
Meningioma	43	27	70 (25.0%)
Neuroma	28	21	49 (22.3%)
Ependymoma	9	12	21 (9.5%)
Astrocytoma	9	10	19 (6.8%)
Hemangioblastoma	5	7	12 (4.3%)
Chondroma	5	6	11 (3.9%)
Reticulosarcoma	7	4	11 (3.9%)
Osteosarcoma	3	6	9 (3.2%)
Myeloma	4	2	6 (2.2%)
Osteoma	3	2	5 (1.8%)
Other	43	24	67 (23.9%)
Total	159 (56.8%)	121 (43.2%)	280 (100%)

Based on the ASIA/ISNCSCI scale (2015), the total number of patients with compression myelopathy syndrome (groups A, B, C) was 217 (77.5%) patients i.e., ASIA A-39 (13.9%); B-74 (26.4%), and C-104 (37.1%). The ambulant patients were 63 (21.5%) i.e., ASIA D 47 (16.8%); and E-16 (5.7%).

In 74 (33.6%) cases with extramedullary tumors, underwent an improved newer method of surgical removal of extramedullary tumors.

Multiple comparisons with the patients that had the standard traditional method showed a statistically significant difference in treatment outcomes (*p* = 0.03). Using the newer modified surgical method for extramedullary tumors showed better postoperative outcomes in 44 cases (59.5%) with OR = 1.9; 95% CI 1.1–3.3 (*p* < 0.05) (Table 2).

**Table 2:** Results of surgical treatment of extramedullary tumors.

**Nurick score**	**Advanced (new) method**	**%**	**Traditional method**	**%**	***p*-value**
Grade 1	21	28.4	17	11.6	χ^2^ = 8.7, *p* = 0.03
Grade 2	23	31.1	47	32.3	
Grade 3	22	29.7	64	43.8	
Grade 4	8	10.8	18	12.3	
Total	74	100	146	100	

In 37 (61.7%) cases of intramedullary tumors with an associated intramedullary cyst underwent the newer modified technique. When comparing the two treatment methods for intramedullary tumors, there was no statistically significant difference observed (*p* = 0.15). However, when comparing postoperative Nurick grade 1–2 with grade 3–4, the use of the newer modified method described above demonstrated improvement in the postoperative neurological status in 24 (65%) patients with OR = 3.46; 95% CI 1.2 to 10.3 (*p* < 0.05) (Table 3).

**Table 3:** Results of surgical treatment of intramedullary tumors.

**Nurick scale**	**Advanced (New) method**	**%**	**Traditional method**	**%**	***p*-value**
Grade 1	9	24.3	3	13.0	χ^2^ = 5.9, *p* = 0.15
Grade 2	15	40.5	5	21.7	
Grade 3	9	24.3	8	34.8	
Grade 4	4	10.8	7	30.4	
Total	37	100	23	100	

## Discussion

Surgical removal of spinal cord tumors remains the main, highly effective method of treatment. The technique and tactics of treatment of this pathology were developed in the 1980s of the twentieth century (7, 9–11). Most neurosurgeons recommend total tumor resection in all possible cases (10, 12). However, although postoperative residual tumor is an important predictor of progression, the aggressiveness of resection should be cautiously weighed against the risk of new postoperative neurological deficit (13). Modern approaches to the surgical treatment of spinal cord tumors are aimed at increasing the number of minimally invasive techniques, the use of accurate diagnostic methods, and modern equipment and tools. The use of minimally invasive accesses for the removal of spinal cord tumors has recently been highlighted in a large number of publications (3, 10, 14). Minimal bone resection when accessing the extradural space (hemilaminectomy), according to Yu et al. (14), has several advantages: maximum preservation of the stability of the spine, reduction in the volume of intraoperative blood loss, reduction of muscle dissection, reduction in the duration of the operation and, as a result, a decrease in postoperative pain syndrome and faster physical rehabilitation. The need to use minimally invasive surgical access to maintain the stability of the spine was demonstrated by Byvaltsev et al. (10) in a multicenter study.

In any case, the goal of treating spinal cord tumors is total surgical removal with the possible preservation of orthopedic stability. At the same time, the question of choosing different options for removing tumors, depending on the anatomical location of tumors, remains relevant. When removing intramedullary tumors, the use of microsurgical techniques and minimally invasive accesses can significantly improve the results of surgical treatment and reduce the chances of relapses (6). Anteriorly located extramedullary spinal cord tumors are challenging tasks for the neurosurgeon (10, 15). In some cases, the use of an anterior approach is required for their radical removal. With the ventrally and ventrolaterally located thoracic spine tumors, some authors (16) suggest the use of endoscopic techniques. The use of endoscopy or endoscopic assistance in the resection of spinal cord tumors is a relatively new direction in spinal neuro-oncology.

Intraoperative electrophysiological monitoring is necessary for spinal neurosurgical clinics, especially with intramedullary located tumors. In our study, intraoperative electrophysiological monitoring was used in 12 cases. According to a study of the outcomes of surgical treatment of intradural tumors of the spinal cord conducted by Nambiar and Kavar (12), the frequency of radical resections, according to their data, was 72.3%. A good premorbid clinical stage was the most significant predictor of a positive outcome at discharge and follow-up. The authors concluded that the surgical outcome depends on the premorbid, preoperative, postoperative clinical stage, the degree of radicality of tumor resection, localization, and histological stage of the tumor. This is similar to our findings.

The use of minimally invasive methods for removing spinal cord tumors has advantages in terms of the earlier restoration of lost neurological functions (11, 14). In our opinion, there is a need for a balance between the size of the approach and the possibility of complete removal of the tumor. We, like many authors (2, 6, 10, 17, 18), believe that minimally invasive neurosurgery should primarily be minimally invasive only to neural structures and that tumor resection has to be safe with minimal to no damage to the normal surrounding tissues. The microsurgical technique described for extramedullary tumors emphasizes piece meal tumor removal and starting the initial dissection far from the neural tissue where possible. This in theory reduces the trauma on the neural tissue and allows for clear identification of the tumor, texture and location by the time the neural elements are encountered. This method was superior to traditional tumor removal techniques *p* < 0.05.

Thus, numerous studies (3, 10, 19) have shown that the use of modern operating technologies can avoid instability of the spine, reduce the surgical wound size and infectious complications, the intensity of post-operative pain, the volume of intraoperative blood loss, and the frequency of recurrence of tumor growth.

Syringomyelia associated with intramedullary tumors results from intramedullary tissue damage secondary to hemorrhage or infarction, and resulting from direct secretory ability of intramedullary tumor (20). Some authors consider the presence of a syrinx a favorable prognostic sign because they are commonly associated with non-infiltrative tumors with distinct cleavage planes than more diffuse, infiltrative tumors (21). Whether or not it should be drained remains the question. Some authors have concluded that the syringes resolve spontaneously, we believe the continued pressure on the spinal cord from the syrinx and the fluid collection in the resection cavity can impair the microcirculation and delay recovery in some cases. Other intramedullary cysts like arachnoid cysts have been managed with fenestration with good results (22). Insertion of a syringosubarachnoid shunt as described in the improved method was associated with improved neurological outcome (Nurrick 1 and 2). However, there was no statistically significant difference in overall outcomes.

Tumor resection has to be safe with minimal to no damage to the normal surrounding tissues (13). The microsurgical technique described for extramedullary tumors emphasizes piece meal tumor removal and starting the initial dissection far from the neural tissue where possible. This in theory reduces the trauma on the neural tissue and allows for clear identification of the tumor, texture and location by the time the neural elements are encountered. This method was superior to traditional tumor removal techniques *p* < 0.05.

The limitations of the study includes: low number of patients as this would have affected the significance of our results. We also believe a more comprehensive randomized study would be more informative on the validity of the new improved techniques. The patients' comparisons were not corrected for tumor type and or age but just the procedure performed. This would be very helpful for future to improve the validity of the results.

## Conclusion

Spinal tumors are associated with a high postoperative morbidity. Choosing the optimal surgical approach requires adequate preoperative assessment taking into account multiple patient and tumor factors. We recommend the use of the newer modified techniques described for extramedullary and intramedullary tumors. These procedures were associated with improved postoperative outcome (Nurick 1 and 2) i.e., 59.5 and 64.8% for extramedullary and intramedullary tumors, respectively.

## Data Availability Statement

The original contributions presented in the study are included in the article/supplementary material, further inquiries can be directed to the corresponding author/s.

## Ethics Statement

The studies involving human participants were reviewed and approved by Institutional Review Board at the Republican Specialized Scientific and Practical Medical Center of Neurosurgery. The Ethics Committee waived the requirement of written informed consent for participation. Written informed consent was obtained from the individual(s) for the publication of any potentially identifiable images or data included in this article.

## Author Contributions

KD, GK, and GC: substantial contributions to the conception or design of the work. AS, AM, and GM: acquisition, analysis, and interpretation of data for the work. GK, KD, and GM: drafting and critical revision of the manuscript for important intellectual content. GC, GK, GA, GM, and AM: final approval of the version to be published. All authors contributed to the article and approved the submitted version.

## Funding

The publication was carried out with the support of the Peoples Friendship University of Russia (RUDN) Strategic Academic Leadership Program.

## Conflict of Interest

The authors declare that the research was conducted in the absence of any commercial or financial relationships that could be construed as a potential conflict of interest.

## Publisher's Note

All claims expressed in this article are solely those of the authors and do not necessarily represent those of their affiliated organizations, or those of the publisher, the editors and the reviewers. Any product that may be evaluated in this article, or claim that may be made by its manufacturer, is not guaranteed or endorsed by the publisher.
